# Fracture risk assessment in diabetes mellitus

**DOI:** 10.3389/fendo.2022.961761

**Published:** 2022-09-02

**Authors:** Weiwei Chen, Min Mao, Jin Fang, Yikai Xie, Yongjun Rui

**Affiliations:** ^1^ Department of Endocrinology, Wuxi No.9 People’s Hospital Affiliated to Soochow University, Wuxi, China; ^2^ Department of Orthopeadics Surgery, Wuxi No.9 People’s Hospital Affiliated to Soochow University, Wuxi, China

**Keywords:** bone mineral density, bone turnover markers, diabetes mellitus, microindentation, osteoporotic fractures, quantitative computed tomography, trabecular bone score

## Abstract

Growing evidence suggests that diabetes mellitus is associated with an increased risk of fracture. Bone intrinsic factors (such as accumulation of glycation end products, low bone turnover, and bone microstructural changes) and extrinsic factors (such as hypoglycemia caused by treatment, diabetes peripheral neuropathy, muscle weakness, visual impairment, and some hypoglycemic agents affecting bone metabolism) probably contribute to damage of bone strength and the increased risk of fragility fracture. Traditionally, bone mineral density (BMD) measured by dual x-ray absorptiometry (DXA) is considered to be the gold standard for assessing osteoporosis. However, it cannot fully capture the changes in bone strength and often underestimates the risk of fracture in diabetes. The fracture risk assessment tool is easy to operate, giving it a certain edge in assessing fracture risk in diabetes. However, some parameters need to be regulated or replaced to improve the sensitivity of the tool. Trabecular bone score, a noninvasive tool, indirectly evaluates bone microstructure by analyzing the texture sparsity of trabecular bone, which is based on the pixel gray level of DXA. Trabecular bone score combined with BMD can effectively improve the prediction ability of fracture risk. Quantitative computed tomography is another noninvasive examination of bone microstructure. High-resolution peripheral quantitative computed tomography can measure volume bone mineral density. Quantitative computed tomography combined with microstructure finite element analysis can evaluate the mechanical properties of bones. Considering the invasive nature, the use of microindentation and histomorphometry is limited in clinical settings. Some studies found that the changes in bone turnover markers in diabetes might be associated with fracture risk, but further studies are needed to confirm this. This review focused on summarizing the current development of these assessment tools in diabetes so as to provide references for clinical practice. Moreover, these tools can reduce the occurrence of fragility fractures in diabetes through early detection and intervention.

## Introduction

Diabetes mellitus (DM) affects millions of people as a global debilitating, chronic, epidemic disease. According to an assessment by the International Diabetes Federation (1), 451 million people (aged 18–99 years) worldwide had diabetes in 2017, which is expected to reach 693 million in 2045. Diabetes-related chronic heart, brain, kidney, eye, and nerve complications have a high rate of disability and mortality, reducing the quality of life and placing a huge economic burden on society (2–4). Osteoporotic fractures are also a public health problem with the aging of the population. However, the adverse effects of diabetes on bone are often underestimated or even ignored in clinical practice. In recent years, increasing evidence shows that diabetes is an independent risk factor for osteoporosis (5). Osteoporosis is defined as a systemic skeletal disease characterized by low bone mass and microarchitectural deterioration of bone tissue, with a consequent increase in bone fragility and susceptibility to fracture (6). Increased bone fragility in patients with diabetes is regarded as a common and serious complication of diabetes (7). Studies have shown that both type 1 DM (T1DM) and type 2 DM (T2DM) have a correlation with a higher fracture risk (8). The fracture risk of T1DM is three times as high as that in the general population (9), and it occurs 10–15 years earlier than that in nondiabetic individuals (10). A meta-analysis including 30 epidemiologic studies reported a significant association between T2DM and increased risk of overall fracture [summary relative risk =1.05, 95% confidence interval (CI): 1.04–1.06] (11). Recently, a population-based cohort study of 6,548,784 Korean individuals including 2418 patients with T1DM and 506,208 patients with T2DM demonstrated that fracture risk was higher in patients with diabetes than in nondiabetic individuals for all measurement sites (12). Moreover, diabetes combined with a fracture leads to reduced activities and prolonged hospital stay, further affecting the quality of life of patients, increasing the rate of disability and mortality, and bringing a huge economic burden to patients themselves and the development of society. Therefore, identifying patients with diabetes combined with a high fracture risk early is crucial.

However, the pathophysiological mechanism of fractures in patients with diabetes is complex and multifactorial. Although bone mineral density (BMD) has a correlation with bone strength, the contribution rate of BMD to bone strength is only approximately 70% (13, 14). The aforementioned manifestation is more pronounced in T2DM, as the BMD of patients with T2DM is usually normal or even higher; also, the fracture risk is higher than that in nondiabetic populations (15, 16). This suggests that other factors, such as impaired quality caused by accumulation of glycation end products, low bone turnover, and microstructural changes, which make adverse effect on mechanical properties of bone tissue, can affect the overall bone fragility and lead to the increased fracture risk in patients with diabetes (7). Moreover, extrinsic factors, such as hypoglycemia caused by treatment, diabetes peripheral neuropathy, muscle weakness, visual impairment, and some hypoglycemic agents including thiazolidinediones, SGLT2i, and insulin, also increase the fracture risk (17–19). This review aims to summarize the diabetes-related fracture risk assessment tools and discuss their advantages disadvantages. Furthermore, the review might provide a reference for the risk assessment of osteoporotic fractures in patients with diabetes.

## BMD

BMD is an important tool for assessing bone mass in clinical practice. It is traditionally thought that DXA-measured BMD is the gold standard for assessing osteoporosis. It can be used for diagnosing osteoporosis, fracture risk prediction, and drug efficacy assessment (20). Its advantages are simplicity, convenience, noninvasion, low cost, and low radiation, while its disadvantage is that DXA is a two-dimensional projection technology that can only measure the area bone density. Therefore, it cannot reflect the complex 3D bone characteristics. Measurements are mainly made at the axial skeleton, lumbar spine, and proximal femur, which are susceptible to lumbar degeneration and abdominal aortic calcification. Moreover, the measurement results of different DXA machines cannot be compared with each other without transverse quality control.

Although BMD is an important tool for assessing bone mass, the assessment of fracture risk in patients with diabetes remains controversial, especially in patients with T2DM. Studies have consistently shown lower BMD in patients with T1DM compared with controls without diabetes (9, 21). However, the fragility fracture risk in patients with T1DM is much higher than the value-at-risk predicted by the DXA method (22, 23). Additionally, the changes in the BMD of patients with T1DM of different sexes and ages are inconsistent with those of nondiabetic individuals (23). This indicates that the BMD cannot be used to generalize fracture risk in patients with T1DM. Although different studies showed discrepant results, most reported that T2DM had higher BDM than control (16, 24, 25). Nonetheless, increasing evidence indicates that patients with T2DM have an increased fracture risk. A meta-analysis including 16 studies involved 1,758,225 participants. It reported 59,909 nonvertebral fracture events and 6430 vertebral fracture events. The regulated relative risk of T2DM and nonvertebral fractures in men was 1.20 (95% CI: 1.09–1.31) (26). Similarly, another meta-analysis including observational studies showed that adults with diabetes had a higher risk of fractures for both hip fractures (RR 4.93, 3.06–7.95 in T1DM and RR 1.33, 1.19–1.49 in T2DM) and nonvertebral fractures (RR 1.92, 0.92–3.99, in type 1 and RR 1.19, 1,11–1.28 in type 2) compared with adults without diabetes (27). In conclusion, BMD alone may underestimate the fracture risk in patients with diabetes. As BMD measured by DXA can only assess changes in bone mass, only approximately 70% of changes in bone strength can be reflected. Hence, it is almost impossible to determine bone microstructure related to bone quality, whose damage is closely connected with the assessment of high fracture risk (28).

## Fracture risk assessment tool

The fracture risk assessment tool (FRAX) is a web-based assessment tool developed and recommended by the World Health Organization for predicting the osteoporotic fracture risk (http://www.shef.ac.uk/FRAX). The model is established mainly based on clinical risk factors (including age, gender, height, body mass, previous fractures, parental hip fractures, smoking, glucocorticoids, rheumatoid arthritis, secondary osteoporosis, and excessive alcohol consumption) and femur and neck BMD (if any or no) to predict the probability of hip fracture and major osteoporotic fracture (centrum, forearm, hip, or shoulder) over the next 10 years (29). It is simple and convenient for FRAX to predict fracture risk, but with certain limitations. First, FRAX is not applicable for people who have been diagnosed with osteoporosis, have had fragility fractures, or have received effective anti-osteoporosis therapy. Second, differences exist in prediction ability between different regions and ethnic populations. Third, the major clinical risk factors for osteoporotic fractures included in FRAX are incomplete, with a lack of specific quantification.

It has been found in clinical practice that patients with diabetes have a higher fracture risk than the general population. However, it is not considered as a clinical risk factor by FRAX, which may influence its application in the population with diabetes. A clinical study with large samples in Canada (including 3518 patients with diabetes and 36,085 nondiabetic individuals) showed (30) that patients with diabetes had a higher fracture risk than the nondiabetic population, but the prediction ability of patients with diabetes scored by the FRAX was lower than that of the nondiabetic population. Besides, a population-based, multicenter, and cross-sectional study on postmenopausal osteoporosis in Japan indicated that patients with T2DM had an increase in the mean risk of severe osteoporotic fractures when using the FRAX without the BMD for scoring. Nonetheless, the FRAX major osteoporotic fracture risk without the BMD did not correctly indicate current bone fragility in middle-aged Japanese women with T2DM (31). Another study (32) included 566 women aged 40–90 years, with different glycometabolic states [normoglycemia, impaired fasting glucose (IFG), and diabetes]. It indicated that women with diabetes compared with women who had normoglycemia or IFG had a higher FRAX score for both major osteoporotic fracture and hip fractures, when the BMD was not included. When the BMD was included, no difference was found. Whether the results of FRAX risk assessment in patients with diabetes are reliable needs further validation, despite the potential role of FRAX in predicting the fracture risk in patients with T2DM. Moreover, the current study results on whether BMD impacted the FRAX score of patients with diabetes were not consistent; therefore, the prospective clinical study validation using large samples is further required.

Despite the limitations of using FRAX to assess the fracture risk in patients with diabetes, the FRAX score is parallel with the fracture risk. The following methods may be useful to enhance the ability to predict the fracture risk in patients with T2DM: The FRAX score is combined with the trabecular bone score (TBS) value, or rheumatoid arthritis is replaced with diabetes (33), or the patient’s age is increased by 10 years when calculating, or the T-value of BMD is regulated appropriately, such as minus 0.5 (34). According to the results from a large Canadian study in the Manitoba cohort (35) that each of the proposed methods of FRAX adjustment was found to improve performance, though no single method was optimal in all settings in T2DM. The perspective from an interdisciplinary expert panel proposed (36) that in T2DM the stratification of fracture risk and treatment thresholds should be mainly based on the presence of a previous fragility fracture and on the individual risk profile, with the inclusion of the additional T2DM related risk factors. Then, referring to the published recommendations in 2018 (37), the related risk factors for fractures in diabetes are outlined in the **Table 1**.

**Table 1:** Risk factors for fractures in diabetes.

**Common risk factors**
FRAX CRF*
Low BMD
Recurrent falls
**Diabetes -specific risk factors**
Diabetes duration>10 years
Diabetes medication: insulin, TZDs, possibly SGLT2 inhibitors
The presence of one or more chronic T2DM complications**
HbA1c > 8% for at least 1 year***

FRAX, fracture risk assessment tool; CRF, clinical risk factor; BMD, bone mineral density; TZD, thiazolidinedione; SGLT2, sodium-glucose cotransporter 2; T2DM, type 2 diabetes mellitus; Hb1Ac, glycated hemoglobin A1c.

*Age, sex, weight, height, previous fracture, family history of hip fracture, current smoking, glucocorticoid, rheumatoid arthritis, alcohol, BMD;

**Microvascular complications: peripheral and autonomic neuropathy, retinopathy, nephropathy;

***Irrespective from disease duration, treatment, or the presence of complications.

## Trabecular bone score

The TBS is a noninvasive tool for assessing the effect of bone microstructure through the analysis of trabecular bone on texture sparsity using special computer software (TBS iNsight) based on the pixel gray variability of spine DXA images (38). Consequently, it has the same interest areas as DXA that assesses BMD, which does not require additional medical equipment or tests. Especially for patients with the same BMD, the differences in bone microstructure and bone quality can be better displayed, thus making up for BMD deficiency in assessing bone quality. Combined with BMD, the ability to predict the fracture risk can be effectively enhanced. Nonetheless, the TBS is not obtained by the physical measurement of bone tissue directly, but it is an overall score calculated after transforming the three-dimensional structure projection of bone tissue into a two-dimensional plane. The calculated TBS value represents the average level of lumbar spines from the first to the fourth (39, 40). Hence, it is only a macro-indicator to assess bone microstructure that cannot reveal specific trabecular bone structures. Also, it cannot assess bone microstructures of other parts except for the lumbar spine. At present, the WHO has not defined the standard reference range of TBS. The clinical recommendations of the TBS reference range for postmenopausal women are as follows: TBS value ≥1.350 is normal, indicating that the bone microstructure is dense and tough, and the connection between trabecular bones is close, with small gaps. A TBS value between 1.200 and 1.350 indicates partial degeneration of bone microstructure, poor connection between trabecular bones, larger gaps, and increased bone fragility. A TBS value ≤1.200 indicates that the bone microstructure degenerates seriously, and the connection between trabecular bones deteriorates further (41).

A large number of clinical studies have confirmed that the TBS of patients with diabetes is lower than that of nondiabetic individuals (42–45). Recently, a meta-analysis involved 35,546 women and 4962 men aged 30 years and older suggested that patients with diabetes had a significantly lower TBS than those without diabetes, with the standardized mean difference being –0.31 (95% CI, –0.45 to –0.16) (46). Moreover, a cross-sectional study in which 119 T1DM (59 males, 60 premenopausal females; mean age 43.4 ± 8.9 years) and 68 healthy controls were analyzed suggested that TBS values were significantly lower in T1DM with prevalent fractures (47). Another observational study including 137 patients with T2DM showed that the TBS negatively correlated with body mass index, waist circumference, HOMA-2IR index, and relative fat mass but positively correlated with the lumbar spine BMD. Other studies showed that the TBS negatively correlated with HbA1c, fasting plasma glucose level, and fasting insulin level (48, 49). As a result, multiple previous studies showed that the TBS was more advantageous in assessing the fracture risk in patients with diabetes, especially T2DM. The TBS can identify the fracture risk in patients with diabetes having normal or increased BMD but changes in bone quality or bone microstructures. The TBS can be used as a supplementary tool for BMD assessment on the fracture risk of patients with diabetes, providing strong evidence for the prevention and treatment of bone-related complications in these patients. However, clinically, the relevant study participants for predicting the fracture risk in patients with diabetes according to the TBS are mostly postmenopausal women, but the study data for adult men and premenopausal women is limited. Whether the TBS can be effectively and accurately applied to assess the osteoporosis risk in such populations still needs more data for further verification. Besides, the increased fracture risk in patients with diabetes cannot be fully explained by the differences in the TBS, and the specific mechanisms need further exploration.

## Quantitative computed tomography

Quantitative computed tomography (QCT) is a method for measuring human BMD based on clinical CT scan data through QCT body model calibration and professional software analysis, which accurately converts CT values of scanned images into the equivalent density of hydroxyapatite. Thus, it is simple, convenient, and noninvasive. While QCT uses CT three-dimensional volume data for analysis, it measures the true volumetric BMD (vBMD). Moreover, it can assess cortical and cancellous bones separately. The interest areas of cancellous bones outlined by QCT on CT images are not influenced by spinal degeneration, body weight, and vascular calcification, and are highly sensitive to subtle changes in bone mass (50, 51). Since the metabolic activity of spinal cancellous bones is approximately eight times that of cortical bones, age-related or treatment-related changes in BMD measured by QCT are more sensitive than the changes in BMD of the entire centrum (cortical bones + cancellous bones) measured by DXA (52). In addition, as CT technology is applied widely in clinical practice and CT scanning of different parts can be performed simultaneously, it can not only meet the needs of clinical routine imaging diagnosis but also measure the BMD on the QCT workstation without increasing the radiation dose. HR-pQCT can be used to image and quantify the volumetric BMD and bone microarchitecture, including cortical porosity, at a low radiation dose (50). Using QCT-based FEMs, a variety of biomechanical parameters can be generated, the bone strength of different parts can be accurately predicted, and the geometric shape, structure, and various mechanical properties of bone can be reflected (53). Therefore, QCT can better reflect the changes in bone strength compared with measuring the BMD by the dual x-ray absorptiometry.

Many studies used QCT and HR-pQCT to assess vBMD and bone microstructure changes in patients with diabetes. However, whether they can predict the fracture risk in patients with diabetes has not been confirmed. The standard sites for predicting fractures by HR-pQCT are distal radius and distal tibia (54). Recently, a cross-sectional study including 59 patients with T1DM and 77 nondiabetic controls showed that patients with T1DM had the lower cortical thickness and lower cortical vBMD at the ultra distal tibia. Bone strength and bone stiffness at the tibia, determined by homogenized finite element modeling, significantly reduced in patients with T1DM compared with controls (55). This study also found that diabetic neuropathy was the determinant of the aforementioned changes, resulting in an increase in fracture risk. Coincidentally, another large, community-based study of elderly patients with T2DM (56) found there were differences in decreased cortical density and increased cortical porosity at the tibia between patients with T2DM and non-T2DM individuals. It was speculated that the decrease in the vBMD and changes in the bone microstructure in the distal tibia might be connected with high fracture risk in patients with diabetes. In contrast, a recent systematic review and meta-analysis showed that the non-weight-bearing distal radius was a more preferable site than the distal tibia for fracture prediction (57). It was speculated that the non-weight-bearing distal radius might be related to microcirculation disorder in patients with diabetes, which was similar to the neuropathy with length dependence. HR-pQCT is expected to be a powerful tool for assessing the fracture risk in patients with diabetes. However, patients with diabetes of different types and ages may have different manifestations. Most of the current studies are limited to cross-sectional observational studies. Consequently, a large number of prospective studies are needed to further verify the correlation between the aforesaid changes in bone parameters and the fracture risk in patients with diabetes.

## Microindentation

Reference point indentation (RPI) is a new technique that has emerged in recent years to directly assess the mechanical properties of bones at the tissue level (58). Two RPI devices have so far been used: Biodent device and OsteoProbe device. The latter is a handheld device. The previous literature suggested that the former be named as cyclic reference point microindentation (CMI) and the latter be named as impact microindentation (IMI) to unify the names (59). Both methods use stainless steel probes with spherical tips (with radii of 2.5 μm and 10 μm, respectively) to press into the bones. The mechanism of action is that the indentation on the bone surface leads to the separation of mineralized collagen microfibers and microcracks. The deeper the probe is pressed into the bone surface, the less the bone tissue can resist mechanical pressure. CMI is mainly used for laboratory tests on *in vitro* samples or animals. As this review mainly focused on the clinical assessment of fracture risk in patients with diabetes, the application methods and parameters of CMI were not repeated here. IMI, a handheld device, is pressed on the bone surface until a force of 10 N is reached, at which time the device generates an additional 30-N impulse force (60). The depth of the probe at the time 10 N is reached is used as the “reference point,” and the distance the probe moves between 10 N and 40 N is measured as an indentation distance increase (IDI). This IDI is normalized to a poly(methyl methacrylate) (PMMA) (plastic) standard, and the device outputs a variable defined as bone material strength index (BMSi), which is a ratio of distances, derived from 100 times the ratio of PMMA IDI to sample IDI. Higher BMSi indicates better mechanical properties of bone tissue. The recommended standard operating procedure has been published (59). The BMSi is associated with the previous fractures and hip fracture risk. This was indicated by a cross-sectional study on men from the Geelong Osteoporosis Study (61). Recently, a systematic review of clinical studies using IMI in humans suggested that the value of BMSi was used not only in evaluating bone fragility but possibly also in the follow-up, particularly for patients with potentially underestimated fracture risk (62). The application of this technique for assessing the properties of bone materials is less invasive, simple, safe, and radiation-free. However, it has not been widely used in clinical practice and is only limited to the tibia. Whether it can reflect the properties of bone materials in the whole body needs further investigation.

Microindentation technique has been applied in clinical studies to assess the properties of bone materials in patients with diabetes. In 2019, a Spanish cross-sectional study (63) of 45 premenopausal women with T1DM and 21 healthy women found no differences in the BMSi or BMD between patients with T1DM and controls. The study speculated that the aforementioned finding might be correlated with intensive insulin therapy. This study was the first known investigation to assess the BMSi in patients with diabetes, which included premenopausal women without a history of osteoporosis or fracture. A cross-sectional study on men with T1DM from Norway in 2021 showed that the BMSi was lower in patients with T1DM compared with healthy age-matched men (64). In 2016, a population-based study (65) on elderly women with diabetes in Sweden indicated that the BMD of patients with T2DM was higher, but the BMSi was lower than that of controls (74.6 ± 7.6 vs 78.2 ± 7.5, *P* < 0.01). Meanwhile, women with T2DM performed clearly worse in measures of physical function. Reduced BMSi and impaired physical function might explain the increased fracture risk in T2DM. Additionally, the Geelong Osteoporosis Study on participants with different glycometabolic states, including 340 men aged 33–96 years, showed that when considering the glycemic status as a binary variable, men with T2DM had a lower mean BMSi compared with those without T2DM (normoglycemia and IFG combined), and this difference in the BMSi was independent of femoral neck BMD (66). Therefore, the BMSi can reflect the changes in the bone quality of patients with diabetes. Despite no clear relationship between BMSi and bone mass, IMI could be used as an additional tool to DXA-measured BMD in assessing bone health. Nevertheless, prospective studies using large samples with standard procedures are lacking at present.

## Bone histomorphometry

Bone histomorphometry mainly uses two-dimensional microscopic images of bone tissue sections for analysis. Through analysis, measurement, and calculation for the target images, the measurement parameters of bone tissue structure can be obtained, including static and dynamic parameters of the bone (67). Static parameters are mainly used to quantitatively describe the characteristics of bone structure at a specific time point, including the thickness, volume, and surface area of bone structure, as well as the area and volume of osteoid tissue. Dynamic parameters are mainly used to quantitatively analyze the status of bone formation and bone resorption at a certain time. The reasons for the changes in static parameters are explained by describing the rate of bone surface mineralization. In the application process, static and dynamic parameters are often combined to comprehensively analyze and judge the biological characteristics of bone. It can not only qualitatively analyze the morphological and structural changes in bone tissue but, more importantly, quantitatively analyze the characteristics of bone microstructure, such as the thickness and porosity of bone cortex, the area and thickness of trabecular bone, the number of connecting points of trabecular bone, and the rate of new bone formation. The biological properties of bone can be measured on a comparative objective basis (67, 68). Nonetheless, its clinical application is limited due to the cumbersome preparation and technique and the invasiveness of this examination. It is now commonly used for exploring the causes and pathogenesis related to osteoporosis based on animal or clinical trials, especially in the research and development of prevention and treatment drugs (69). The micro-CT measurements were performed on intact bone biopsies to capture the differences in three-dimensional (3D) trabecular bone architecture and volumetric density (70).

Currently, the use of bone histomorphometry in patients with diabetes is limited. An early study in 1995 on bone histomorphometry in patients with T1DM and T2DM showed that patients with diabetes had a low rate of bone formation; including two patients with T1DM and six with T2DM who had bone biopsies (71). Another case–control study included 18 patients with T1DM, among which 5 had a history of fragility fractures. Compared with the healthy age- and sex-matched nondiabetic controls, no significant differences in histomorphometric or micro-CT measurements were found (72). However, a discrepancy in structural and dynamic trends was noted between patients with diabetes and fractures and those with diabetes but no fracture. The results of the aforementioned two studies were inconsistent, possibly because the latter included patients with diabetes having good blood glucose control and relatively short disease course, as well as those without severe diabetic complications, and these speculated factors might directly or indirectly impact the bone status. The invasiveness and complexity of bone histomorphometry have limited its application in clinical practice, although it is the gold standard for assessing bone metabolic status. It is necessary to not only improve the technique and simplify the procedure but also conduct bone histomorphometry studies using larger samples in different diabetic populations to guide the assessment and treatment of fracture risk in patients with diabetes.

## Bone turnover biomarkers

Bone turnover biomarkers (BTMs) are metabolites or enzymes generated during bone turnover; they are divided into bone formation indicators and bone resorption indicators (73). The former reflects osteoblast activity and bone formation status, including total alkaline phosphatase (ALP), bone-specific alkaline phosphatase (b-ALP), procollagen type 1 N-terminal propeptide (P1NP), procollagen type 1 C-terminal propeptide (P1CP), and osteocalcin (OC). The latter represents osteoclast activity and bone resorption levels, mainly including hydroxyproline (HOP), pyridinoline (Pyr), deoxypyridinoline (DPD), carboxy-terminal cross-linked telopeptide of type 1 collagen (CTX), amino-terminal cross-linked telopeptide of type 1 collagen (NTX), and tartrate-resistant acid phosphatase 5b (TRAP5b). Furthermore, multiple important cytokines are involved in the bone turnover regulation by regulating osteogenic or osteoclast activity, such as osteoprotegerin (OPG), RANKL, sclerostin, and interleukin, all of which can be considered as regulatory factors for bone turnover (74). All these biomarkers can be obtained from blood or urine specimens; thus, the tool is simple, easy to operate, and reproducible. However, bone formation is coupled to bone resorption, which is precisely regulated by multiple factors such as endocrine hormones, mechanical stress, and drugs. Hence, the results, which are highly variable, should be carefully interpreted in clinical practice based on the actual situation of the individual (73). Bone turnover is a process of bone modeling and remodeling. Hence, BTMs can reflect the metabolic rate of bone, whose metabolic imbalance is the key pathophysiological mechanism underlying various bone diseases. Accelerated bone turnover, especially enhanced bone resorption, can cause a decrease in BMD and the breakage of bone microstructure; thus, the bone strength is reduced while the fracture risk increases (75). This is particularly obvious in postmenopausal women (76). Previous studies also indicated that bone metabolic biomarkers were independent factors for predicting new fractures regardless of previous BMD and fracture history (77). Hence, these markers are of great value in fracture risk assessment.

Abundant evidence demonstrates that bone turnover is low in patients with diabetes (78–80). In recent years, a meta-analysis involving 66 studies showed that CTX, OC, and P1NP levels in patients with diabetes were lower than those in controls, while the levels of sclerostin and OPG were higher than those in controls (81). Moreover, a cross-sectional study including 101 patients with T1DM and 96 with T2DM (patients in both case and control groups had diabetes, and they were grouped according to whether they had a vertebral fracture or incident fracture) demonstrated that sclerostin had different effects on the bone turnover of patients with T1DM and T2DM, which negatively correlated with the fracture occurrence in patients with T1DM, but positively correlated with the fracture occurrence in patients with T2DM (82). Nonetheless, the sample size of this study was small, including 21 patients with T1DM combined with fracture and 13 patients with T2DM combined with fracture. Thus, sclerostin seemed to have a predictive value for fracture risk in patients with diabetes. However, prospective studies with large samples are still needed for verification. Recently, a case–cohort study, including 223 participants who experienced incident fractures of the hip, clinical spine, or distal forearm and the subcohort comprising 508 participants randomly assigned according to 3 different blood glucose states (normoglycemia, prediabetes, and T2DM) at baseline, showed that the fracture risk increased with higher CTX, OC, P1NP levels in nondiabetic individuals, but did not increase in patients with T2DM (83). Thus, it was concluded that BTMs did not predict incident fracture risk in patients with T2DM but were modestly associated with fracture risk in nondiabetic individuals. To sum up, whether BTMs can predict fracture risk in patients with diabetes is still controversial. It might be related to the wide variety of BTMs, inconsistent statistical methods in various studies, and many confounding factors. Any single bone turnover biomarker cannot indicate the fracture risk in patients with diabetes. Therefore, BMD should be combined to make a comprehensive evaluation. Further studies should search for an objective, comprehensive, and easily accessible biomarker for indicating the fracture risk in patients with diabetes.

## Discussion

As is discussed in the introduction, substantial evidence suggests that diabetes mellitus is associated with an increased risk of fragility fractures. Albeit each of the discussed methods above (**Table 2**) including BMD、FRAX、TBS、HR-pQCT、Microindentation、BTMs、bone histomorphometry is a powerful tool to evaluate the osteoporotic fracture risk in patients with diabetes, no single method is optimal in all settings, particularly in T2DM, which may be attributed to complex pathophysiological mechanisms.

**Table 2:** Summary of fracture risk assessment methods and their respective advantages and disadvantages.

Fracture risk assessment methods	Advantages	Disadvantages
BMD measured DXA	Simplicity, noninvasion, low cost, low radiation	Area bone density of the axial skeleton, lumbar spine, and proximal femur; be susceptible to lumbar degeneration and abdominal aortic calcification; the measurement results of different DXA machines without transverse quality control cannot be compared with each other.
FRAX	Simplicity, noninvasion, low cost, low radiation	Not apply to people who have been diagnosed with osteoporosis, have had fragility fractures, or have received effective anti-osteoporosis therapy; exist differences in prediction ability between different regions and ethnic populations; incomplete about the major clinical risk factors for osteoporotic fractures; lack of specific quantification range.
TBS	Noninvasion; not require additional medical equipment or tests; make up for BMD deficiency in assessing bone microstructure and bone quality	A macro-indicator to assess bone microstructure indirectly; only assess lumbar spines; no special reference range excepting in postmenopausal women.
QCT	Simplicity, noninvasion; highly sensitive; be able to measure vBMD, assess cortical and cancellous bones separately; not influenced by spinal degeneration, body weight, and vascular calcification;	Expensive, unavailable, relatively high radiation, the estimate of cortical porosity may be difficult in areas with thin cortices and/or high trabecular bone volume.
Microindentation	Directly assess the mechanical properties of bones at the tissue level; simplicity, safety, free-radiation, less invasion	Not been widely used in clinical practice, only limited to the tibia
Bone histomorphometry	Obtain static and dynamic parameters of the bone, gold standard for assessing bone turnover	Invasion, complexity
BTMs	Reflect bone formation and bone resorption; simplicity, easy to operate, and repeatability	Affected by many confounding factors, the wide variety

BMD, bone mineral density; DXA, dual x-ray absorptiometry; FRAX, fracture risk assessment tool; TBS, trabecular bone score; QCT, quantitative computed tomography; vBMD, volumetric BMD; BTMs, bone turnover biomarkers.

Insulin is anabolic for bones, and insulin-like growth factor-1 (IGF-1) receptor plays a critical role in the execution of the anabolic effect of insulin on osteoblasts (84). Patients with T1DM and T2DM both manifested by hyperglycemia have different insulin level, that the former is mainly insulin-deficient and the latter is characterized by insulin resistance. As a result, the mechanism of increased bone fragility in T1DM and T2DM has something in common while have something in difference. Insulin-deficient conditions in T1DM are typically associated with low levels and/or action of IGF-1, which have an adverse effect on osteoblasts during growth and can result in low peak bone mass at an early age (85–87). This is consistent with the results that lower BMD in T1DM than general population confirmed by previous studies. Although insulin resistance is the most significant feature of T2DM, which seem to partly explain the normal or higher BMD in these populations, it can also make bone quality worse by the following means. First, hyperglycemia leads to osteoblast resistance to the actions of IGF-1 (88). Second, a high level of AGEs reduces the stimulatory effect of IGF-1 on osteoblasts (89). On the other hand, higher bone resorption and adipogenesis in the state of insulin resistance are induced by loss of Dock 7 protein and silencing of Thy-1 expression, which contributes to loss of bone mass (90). Moreover, normal calcium and phosphorus metabolism of bone tissue may be disrupted by long-term hyperglycemic stimulation and insulin resistance to promote a chronic low-grade inflammatory response. It results in bone remodeling obstruction and bone microstructure deterioration, which may ultimately cause a decrease in bone quality and an increase in fracture risk in patients with DM (91). In addition, hypogonadism induced by obesity and insulin resistance has also lead to low bone mass (92). To summarize, both insulin deficiency and insulin resistance are associated with low bone mass. BMD is not decreased in T2DM which is related to hyperinsulinemia secondary to insulin resistance. Although patients with diabetes from different areas have somewhat different pathophysiological features, such as not being so obese or less insulin resistant, the role of insulin resistance in determining fracture risk is still under debate (93). Nevertheless, patients in T2DM are prone to greater fracture risk than general population, which may correlate with the damage of bone quality and other mechanisms. In fact, BMD cannot completely capture the compromised bone quality in diabetes.

As noted already in QCT section, the cortical porosity in T2DM is higher than in non-T2DM individuals (56). Interestingly, another cross-sectional observational study (94) including 171 T2DM patients (mean age, 68.8 years) and 108 age-matched non-diabetic controls found that T2DM patients with clinically significant peripheral vascular disease, assessed by transcutaneous oxygen tension(TcPO2)≤ 40 mm Hg-a measure of microvascular blood flow, had higher (+21.0%, *p* = 0.031) cortical porosity at the distal tibia in comparison to controls. Collectively, peripheral vascular disease in diabetes may be the potential mechanism leading to increased cortical porosity, as a result to impair bone quality. Moreover, recent studies found that patients with microangiopathy had a higher rate of osteopenia and osteoporosis than patients with diabetes without microangiopathy (95). Furthermore, diabetes-related vascular changes (cortical microangiopathy) had been postulated as the reason behind the poor cortical bone quality in diabetic patients with a fracture (96, 97). Therefore, it suggests that diabetes combined with osteoporosis may be another manifestation of microvascular diseases, which may be related to the decrease in the levels of bone resorption markers and osteocalcin. On the other hand, in the Maastricht Study (98), T2DM patients with HbA1c <7% had superior cortical bone quality than those with poor glycemic control, but no significant relation was found with the microvascular disease. Therefore, the specific mechanism between cortical porosity or microangiopathy and bone quality is still unclear.

In addition, the compromised bone quality are related to the course of diabetes and the glycemic control condition, whose specific mechanism are as follows. First, hyperglycemia acts on osteocytes, osteoblasts, and osteoclasts to reduce bone turnover. In the case of hyperglycemia in patients with diabetes, osteoclast recruitment can be inhibited by reducing the receptor activator for nuclear factor-κB ligand (RANKL) production (99). The bone formation can also be inhibited by increasing sclerostin and Dickkopf-1 (which can inhibit the pro-osteogenic Wnt signaling pathway) (100). Hence, osteocytes do not experience the damage caused by mechanical stimulation, resulting in microtrauma accumulation and thus reducing bone strength. Second, hyperglycemia can also reduce the activity of mesenchymal stem cells, inhibit their differentiation into osteoblasts, and promote their differentiation into adipocytes (101). Third, long-term hyperglycemia leads to the accumulation of advanced glycation end products (AGEs). Besides inhibiting the proliferation and differentiation of osteoblasts, AGEs accumulated in the body can not only promote oxidative stress and produce nonenzymatic crosslinking with type I collagen (the two best-characterized AGEs related to collagen are pentosidine and N-carboxymethyllysine) but also damage bone matrix and increase bone stiffness, increasing the chances of fractures (102, 103). Although some studies indicated that serum or urinary levels of pentosidine correlated with the risk of vertebral fracture in T2DM (104, 105), the feasibility of predicting fracture risk needs to be further confirmed. Recently, some evidences showed that AEGs negatively correlated with BMSi especially in T2DM (94, 106, 107). It supports the hypothesis that AGEs play a potential role in the development of skeletal abnormalities in human T2DM, and the accumulation of AGEs probably contributes to impaired bone material properties. In addition, AGEs can bind to receptor for AGE (RAGE, a transmembrane protein produced by the Agergene) which partly place in the osteoclastic and osteoblastic cell lineages, to regulate bone resorption and bone formation by signal transduction (108, 109). Collectively, hyperglycemia can damage bone quality by regulating bone turnover, stimulating adipogenesis and increasing the accumulation of AGEs.

## Conclusions and prospects

In conclusion, patients with diabetes have a high risk of osteoporotic fractures, which cause great harm. Considering the complex pathophysiological mechanism, it is difficult to identify these patients in early clinical practice. BMD, measured using the traditional DXA and FRAX, tends to underestimate the fracture risk in patients with diabetes, and the TBS can indirectly assess the changes in bone microstructure to make up for the shortcomings of the aforementioned methods. Not only can subtle changes in bone mass be reflected, but the bone microstructure, bone geometric shape, and mechanical properties can be assessed using QCT, QCT-based FEMs, and HR-pQCT. Despite being of great value for fracture risk assessment in patients with diabetes, it has not been widely applied in clinical practice. Microindentation and histomorphometry can directly assess the mechanical properties of bone, but their clinical use is limited due to their invasiveness. By virtue of the diversity of BTMs and the lack of consistent studies, whether they can predict the fracture risk in patients with diabetes remains controversial. To sum up, the aforesaid methods are favorable tools for assessing the fracture risk in patients with diabetes, but none of them can comprehensively and multi-dimensionally assess the fracture risk in such patients. As a consequence, multiple methods should be combined in clinical practice to identify the osteoporotic fracture risk in patients with diabetes early.

Additionally, whether osteoporosis should be listed as a microvascular complication for patients with diabetes, and whether these patients with a high fracture risk can be identified through the early screening for microvascular complications, especially diabetes peripheral neuropathy, needs to be explored in the future. Also, it is expected to be an important method for predicting fractures that the vBMD of distal radius as well as the distal tibia, and the change of bone microstructure are measured by HR-pQCT. However, further prospective studies are needed to verify which site changes are unique to patients with diabetes. The aforementioned views may provide new ideas for the early identification of high fracture risk in patients with diabetes considering the lack of unified screening procedures and diagnostic criteria for patients with diabetes and osteoporotic fractures. It is expected that the high fracture risk in patients with diabetes can be early screened and identified so that the living quality of patients with both diseases can be comprehensively improved through unremitting efforts.

## Author contributions

WC designed and drafted the manuscript. MM, JF, and YX revised the manuscript. YR reviewed this article. All authors contributed to the article and approved the submitted version.

## Funding

This work was provided by Wuxi Top Medical Expert Team of “Taihu Talent Program”.

## Acknowledgments

We thank all members of the department of endocrinology for discussions.

## Conflict of interest

The authors declare that the research was conducted in the absence of any commercial or financial relationships that could be construed as a potential conflict of interest.

## Publisher’s note

All claims expressed in this article are solely those of the authors and do not necessarily represent those of their affiliated organizations, or those of the publisher, the editors and the reviewers. Any product that may be evaluated in this article, or claim that may be made by its manufacturer, is not guaranteed or endorsed by the publisher.
